# A prospective observational study of patient-reported functioning and quality of life in advanced and metastatic breast cancer utilizing a novel mobile application

**DOI:** 10.1007/s10549-020-06082-7

**Published:** 2021-01-11

**Authors:** David Richardson, Lin Zhan, Reshma Mahtani, Lynn McRoy, Debanjali Mitra, Maria Reynolds, Dawn Odom, Kelly Hollis, James A. Kaye, Cheryl Jones, Jeffrey Hargis

**Affiliations:** 1 RTI Health Solutions, 3040 East Cornwallis Road, Post Office Box 12194, Research Triangle Park, NC 27709-2194 USA; 2 Pfizer, Inc, New York, NY USA; 3 University of Miami, Deerfield Beach, FL USA; 4 Northside Hospital Inc, Atlanta, GA USA; 5 Norton Cancer Institute, Louisville, KY USA

**Keywords:** Patient-reported outcomes, Advanced breast cancer, Metastatic breast cancer, Cyclin-dependent kinase 4/6, Palbociclib

## Abstract

**Purpose:**

To assess and describe patient-reported outcomes (PROs) in women with locally advanced/unresectable or metastatic breast cancer (aBC/mBC) with hormone receptor-positive/human epidermal growth factor receptor 2-negative (HR + /HER2 −) status receiving palbociclib combination therapy in a US real-world setting.

**Methods:**

A prospective, noninterventional, multicenter longitudinal study was conducted in US patients initiating treatment with palbociclib combination therapy for HR + /HER2 − aBC/mBC. PRO data (SF-12; CES-D-10; mood; pain; fatigue; interference of aBC/mBC or its treatment on family life, social life, physical activity, energy, and productivity; overall health rating; and quality of life [QOL]) were collected via a custom-developed mobile application at daily, weekly, and cycle-based intervals. Patient medical information (demographics, clinical characteristics, treatment information, and adverse events) was collected from medical records at baseline and at the end of the 6-month follow-up period.

**Results:**

Patients’ general health status (SF-12) remained consistent throughout treatment and was generally consistent with published norms for individuals diagnosed with cancer. The presence of depression (CES-D-10) was low and did not change substantially over time. Mean pain and fatigue scores using an 11-point numeric rating scale were low and remained stable. Patients, on average, reported neutral or positive moods. Patient-reported QOL and overall health was primarily “Good,” “Very good,” or “Excellent.” Findings were consistent regardless of patient experience with neutropenia.

**Conclusions:**

Patients treated with palbociclib, on average, reported consistently low levels of pain and fatigue as well as good QOL and overall health that remained stable throughout the first 6 months of treatment regardless of episodes of neutropenia.

**Supplementary information:**

The online version of this article (10.1007/s10549-020-06082-7) contains supplementary material, which is available to authorized users.

## Introduction

Breast cancer is the most common cancer among women in the United States (US), with an estimated 276,480 new cases occurring in 2020. Although 5-year survival rates are high among patients diagnosed with early-stage breast cancer, only an estimated 28% of patients with advanced breast cancer (aBC) or metastatic breast cancer (mBC) survive for 5 years [1].

Hormone receptor-positive/human epidermal growth factor receptor 2-negative (HR + /HER2 −) breast cancer is the most common subtype, with an estimated annual incidence rate of 87.0/100,000 women during 2013–2017 in the US [1, 2].

Treatment for patients with HR + /HER2 − aBC/mBC had been unchanged for almost two decades prior to the approval of palbociclib, a first-in-class oral cyclin-dependent kinase 4/6 (CDK4/6) inhibitor approved in 2015. Palbociclib is indicated for the treatment of HR + /HER2 − aBC/mBC in combination with an aromatase inhibitor (AI) as initial endocrine-based therapy and in combination with fulvestrant in patients with disease progression following endocrine therapy in postmenopausal women [3]. In the PALOMA trials, palbociclib combination therapy demonstrated significantly improved progression-free survival, tumor shrinkage, and overall clinical benefit compared with endocrine therapy plus placebo and was associated with primarily hematologic adverse events (AEs), including neutropenia, with a low frequency of febrile neutropenia (1.4%) [4–8].

Since approval, palbociclib has been broadly adopted for treating aBC/mBC, and two other CDK4/6 inhibitors, ribociclib and abemaciclib, have subsequently been approved. For HR + /HER2 − aBC/mBC, current indications for ribociclib are combination treatment with (1) an AI as initial endocrine-based therapy in premenopausal, perimenopausal, or postmenopausal women or (2) fulvestrant in postmenopausal women as either initial endocrine therapy or following disease progression on endocrine therapy [9]. Abemaciclib is currently approved for similar indications to those of palbociclib and as monotherapy in patients with HR + / HER2 − aBC/mBC with disease progression following endocrine therapy and prior chemotherapy in the metastatic setting [10]. CDK4/6 inhibitors have become widely used in practice and are considered standard or preferred options for the treatment of women with HR + /HER2 − aBC/mBC in first line as well as later lines of therapy [11, 12].

There is an extensive body of evidence on the efficacy and safety of CDK4/6 inhibitors broadly and a growing body of real-world evidence focusing on palbociclib’s effectiveness specifically, but little information to date is available regarding the day-to-day impact of aBC/mBC and its treatment on health-related quality of life (QOL) in a real-world setting. We sought to capture this information and the impact of neutropenia, the most common AE for palbociclib, on patient-reported, health-related QOL.

## Materials and methods

### Study design

The study was a prospective, noninterventional, multicenter longitudinal study of US HR + /HER2– aBC/mBC patients initiating first-, second-, or third-line treatment with either palbociclib in combination with an AI (P + AI) as initial endocrine therapy^1^ or palbociclib in combination with fulvestrant (P + Ful) after progression on prior endocrine therapy (according to the US Food and Drug Administration–approved indication for palbociclib) or any other approved therapies for aBC/mBC other than palbociclib [13]. This manuscript focuses on only those patients initiating palbociclib combination therapy.

**Fig. 1:**
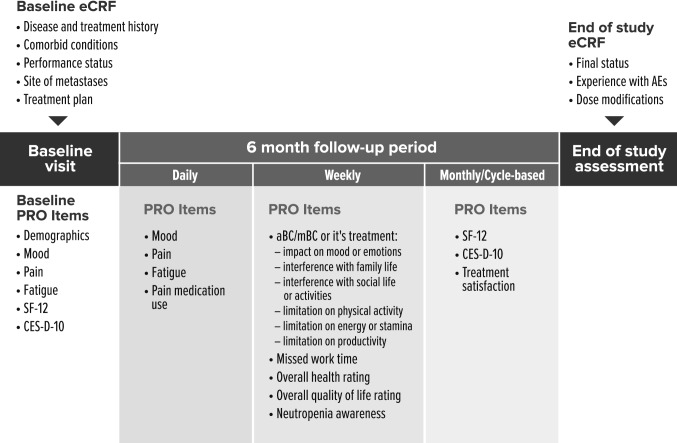
Study design. *aBC* advanced breast cancer, *AE* adverse event, *CES-D-10* 10-Item Center for Epidemiological Studies Depression Scale, *eCRF* electronic case report form, *mBC* metastatic breast cancer, *PRO* patient-reported outcome, *SF-12* 12-Item Short Form Health Survey

Participating investigators screened patients for eligibility, obtained written informed consent, and enrolled patients. Investigators completed electronic case report forms (eCRFs) to capture demographic, medical history, and treatment information at enrollment and recorded interim changes in treatment, clinical outcomes, and AEs for 6 months after enrollment. Enrolled patients were provided access to and trained on the use of a custom-developed mobile application, downloaded to their smartphones, to complete baseline, daily, weekly, and cycle-based patient-reported outcome (PRO) assessments for the 6-month period (Fig. 1). PRO and eCRF data were combined at the patient level.

Data collection for this study occurred over a 33-month period (February 2017–October 2019). The data collection period for an individual patient was approximately 6 months, which could be truncated by treatment switching, patient withdrawal from the study, or death. Follow-up ended if patients discontinued palbociclib combination therapy.

### Eligibility

Eligibility was assessed prior to enrollment during a scheduled visit; any woman who met the eligibility criteria was invited to participate. Eligible women were aged ≥ 18 years with a diagnosis of adenocarcinoma of the breast and evidence of aBC/mBC not amenable to resection or radiation therapy with curative intent, had evidence of HR + /HER2 − tumor based on the patient’s most recent tumor biopsy, and owned or had regular access to a smartphone. Exclusion criteria were as follows: life expectancy < 3 months at the time of diagnosis of aBC/mBC, participation in any interventional clinical trial, and active treatment for other malignancies other than aBC/mBC.

### Endpoints

Study endpoints included PRO assessments (to describe patients’ symptoms, health status, mood, depression, ability to function, overall health, and QOL) and safety information (AEs).

Questionnaires assessing PROs included the 12-Item Short Form Health Survey (SF-12), the 10-Item Center for Epidemiological Studies Depression Scale (CES-D-10), and targeted questions, which were made available to patients via the mobile application downloaded onto their smartphones [14–17]. Patients completed a baseline questionnaire as well as a series of questions at daily, weekly, and cycle-based intervals. Not all measures collected are examined in this manuscript. Standard survey methodological principles were used to draft items [18].

## Measures

### Daily and weekly assessments

Pain and fatigue severity were measured using an 11-point numeric rating scale (NRS); 0 indicated no pain or fatigue, and 10 indicated the worst possible pain or fatigue.

Mood was rated daily on a 7-point scale (“Very sad,” “Sad,” “Discontent,” “Neutral,” “Content,” “Happy,” “Most happy”) and summarized at the weekly level to determine the mean percentage of nonmissing days for each mood level across all patients.

Patients indicated weekly how breast cancer or its treatment interfered with family/social life, productivity, physical activities, and energy on a 5-point scale (“Not at all,” “A little,” “Moderately,” “Quite a bit,” “A great deal”). Additionally, patients rated their health and overall QOL during the past 7 days on a 5-point scale (“Poor,” “Fair,” “Good,” “Very good,” “Excellent”).

### Cycle-based assessments

#### SF-12

The SF-12 is a shortened version of the 36-Item Short Form Health Survey for measuring general health status in study populations [19]. Physical Component Summary (PCS-12) and Mental Component Summary (MCS-12) scale scores were calculated from responses to yield a mean score of 50 and a standard deviation (SD) of 10 in the US population; higher scores represented better health. The PCS-12 and MCS-12 summary measures provide a rough comparison of QOL for patients in this study with the general population and published norms for cancer patients.

#### CES-D-10

The CES-D-10 is a 10-item self-administered questionnaire assessing depression during the last week. The 10 items are rated on a 4-point scale ranging from 0 (< 1 day) to 3 (5–7 days) and summed to produce a total score (range, 0–30). A total score of 10 or more is considered to indicate depression [14].

### Safety endpoints

Safety endpoints included the incidence, severity, and duration of neutropenia events and other AEs occurring during the study. AEs were recorded in the eCRF and included selected AEs of special interest (nonfebrile neutropenia, febrile neutropenia, leukopenia, infection, fatigue, nausea, anemia, stomatitis, headache, diarrhea, thrombocytopenia, constipation, alopecia, vomiting, rash, and decreased appetite), dates of onset and resolution, National Cancer Institute Common Terminology Criteria for Adverse Events toxicity grade, outcome, action taken with treatment, and seriousness. AEs not listed on the eCRF were categorized as “other.”

### Statistical analysis

Analyses were conducted using the full analysis set (FAS), which comprised all patients in the study population with at least baseline mobile app data and data in both the screening and enrollment eCRFs. The SF-12 and CES-D-10 analysis set consists of all patients in the FAS who completed at least one baseline SF-12/CES-D-10 question and at least one postbaseline SF-12/CES-D-10 question.

Descriptive analyses were performed on categorical and continuous endpoints collected or derived from the eCRF and mobile application. Summary statistics were displayed by overall therapy and stratified by line of therapy.

At each cycle, the relationship between all PROs and episodes of neutropenia was explored by comparing patients who did not experience neutropenia during the study (for the cycle-based PROs) and patients who experienced neutropenia at the time point.

Linear mixed-effects models for repeated measures were used to summarize change from baseline values by neutropenia status for the cycle-based PCS-12, MCS-12, and CES-D-10 scores. Fixed effects included treatment, month, treatment by month interaction, neutropenia status at the time point, neutropenia status by month interaction, neutropenia status by treatment interaction, neutropenia status by month by treatment status interaction, and baseline score; a random effect was included for the patient. Least-squares mean estimates and 95% confidence intervals (CIs) for the change from baseline values by neutropenia status were reported for each stratum at each cycle and overall across cycles.

To examine the overall relationship between mood and CES-D-10 score, mixed models for repeated measures were conducted, with CES-D-10 score as the independent variable and a fixed effect for the respective mood question and a repeated effect to account for the correlation within patients. Although mood was available weekly, only the first week of each cycle was used in the analysis to match the recall period of the cycle-based CES-D-10.

As this was primarily a descriptive study, any statistical comparisons are presented at the 2-sided alpha = 0.05 level without adjustment for multiplicity. Any missing data were assumed missing at random. Analyses were conducted using SAS statistical software, version 9.4 (SAS Institute Inc, Cary, North Carolina).

## Results

### Baseline characteristics

This study enrolled 139 evaluable patients from 25 participating sites. Demographic data are shown in Table 1, and clinical data are shown in Table 2. The median (range) age of patients was 60 (34–82) years; 83% were white, 9% were black/African American, 5% were Hispanic/Latino, and 1% were Asian. About half of patients (49%) were employed. Sixty-one percent of patients initiated P + AI and 39% initiated P + Ful. Almost all patients (96%) initiated palbociclib at a dose of 125 mg/day. Based on medical records, 22% of patients had Stage IV, de novo, disease at diagnosis. At enrollment, the median duration between mBC diagnosis and study enrollment was 1.0 month. Thirty-nine percent of patients had visceral metastases and 41% had bone-only disease. Most patients had an Eastern Cooperative Oncology Group performance status of 0 (63%) or 1 (24%).

**Table 1:** Patient demographic characteristics

Category	P + AI (n = 85)	P + Ful (n = 54)	Overall (N = 139)
Age, mean (SD), y	59.3 (12.62)	60.3 (10.20)	59.7 (11.72)
Age group, n (%)			
< 55	33 (39)	13 (25)	46 (33)
55 to 64	20 (24)	20 (38)	40 (29)
65 to 74	22 (26)	16 (30)	38 (28)
75 to 84	10 (12)	4 (8)	14 (10)
Missing	0	1	1
Race or ethnic origin, n (%)			
White	70 (82)	46 (85)	116 (83)
Black/African American	7 (8)	6 (11)	13 (9)
Hispanic/Latino	6 (7)	1 (2)	7 (5)
Asian	2 (2)	0 (0)	2 (1)
Other	0 (0)	1 (2)	1 (1)
Education level, *n* (%)			
Less than high school	2 (2)	1 (2)	3 (2)
High school diploma or equivalent	13 (16)	13 (24)	26 (19)
Some college	24 (29)	11 (20)	35 (26)
College degree	25 (30)	19 (35)	44 (32)
Professional or advanced degree	19 (23)	10 (19)	29 (21)
Missing	2	0	2
Employment status, *n* (%)			
Employed full-time	33 (39)	19 (35)	52 (38)
Employed part-time	9 (11)	6 (11)	15 (11)
Retired	33 (39)	21 (39)	54 (39)
Not employed	9 (11)	8 (15)	17 (12)
Missing	1	0	1
Type of insurance, *n* (%)			
Commercial/private	45 (54)	30 (56)	75 (54)
Medicare	30 (36)	21 (39)	51 (37)
Medicaid	6 (7)	2 (4)	8 (6)
Other government-sponsored	2 (2)	1 (2)	3 (2)
Uninsured	1 (1)	0 (0)	1 (1)
Missing	1	0	1

*P* + *AI* palbociclib in combination with an aromatase inhibitor, *P* + *ful* palbociclib in combination with fulvestrant, *SD* standard deviation

Only patients with at least baseline mobile app data and data in both the screening and enrollment electronic case report forms are included in the full analysis set

**Table 2:** Patient disease history and clinical characteristics

Category	P + AI (*n* = 85)	P + Ful (*n* = 4)	Overall (*N* = 39)
Type of breast cancer, *n* (%)			
mBC	84 (99)	53 (98)	137 (99)
aBC	1 (1)	1 (2)	2 (1)
Duration of breast cancer, mo			
Mean (SD)	71.9 (92.37)	87.9 (78.64)	78.1 (87.35)
Median	34	61	56
Min, Max	0, 378	0, 422	0, 422
Stage at initial diagnosis, n (%)			
I	5 (6)	4 (7)	9 (6)
II	19 (22)	18 (33)	37 (27)
III	17 (20)	17 (31)	34 (24)
IV	22 (26)	8 (15)	30 (22)
Unknown	22 (26)	7 (13)	29 (21)
Duration of mBC, mo			
n	84	52	136
Mean (SD)	5.7 (21.94)	21.8 (37.79)	11.9 (29.94)
Median	1	3	1
Min, Max	0, 184	0, 223	0, 223
Missing	0	1	1
Site of metastases,^a^ n (%)			
n	84	51	135
Visceral	33 (39)	20 (39)	53 (39)
Nonvisceral	51 (61)	31 (61)	82 (61)
Bone-only	36 (43)	20 (39)	56 (41)
ECOG performance status,^b^ n (%)			
0	51 (60)	37 (69)	88 (63)
1	20 (24)	13 (24)	33 (24)
2	3 (4)	1 (2)	4 (3)
Unknown	11 (13)	3 (6)	14 (10)

*aBC* advanced breast cancer, *ECOG*, Eastern Cooperative Oncology Group; *mBC* metastatic breast cancer, *NSAID* nonsteroidal anti-inflammatory drug; *P* + *AI* palbociclib in combination with an aromatase inhibitor, *P* + *Ful* palbociclib in combination with fulvestrant, *SD* standard deviation

^a^Liver, brain, lung/pleura, and ovary metastases were categorized as visceral sites. Bone, lymph node, and skin/soft tissue metastases were categorized as nonvisceral sites. Other sites were categorized as visceral or nonvisceral by clinical review. Patients who had metastasis at any visceral site were classified as having visceral metastases; those without metastases at any visceral site were classified as having nonvisceral metastases

^b^No patients with an ECOG performance status of 3 or 4 were reported

### Physician-reported adverse events and experience with neutropenia

A total of 840 AEs were recorded among 96 patients (69%). AEs affecting ≥ 15% of the 139 evaluable patients included neutropenia (45%), fatigue (35%), leukopenia (35%), anemia (17%), and diarrhea (15%). Twelve patients (9%) experienced at least one serious AE (SAE), with a total of 35 SAEs reported. Nine patients (6%) reported other SAEs, and 3 (2%) reported infection. SAEs of febrile neutropenia, leukopenia, nausea, anemia, thrombocytopenia, vomiting, decreased appetite, and occupational exposure occurred in 1% of patients.

A total of 152 neutropenia events were experienced among 62 patients. Twenty-eight patients experienced 1 event; 15, 2 events; and 19, ≥ 3 events (Table 3). Approximately 28% of events were grade 1; 36%, grade 2; and 33%, grade 3. The mean (SD) duration of neutropenia events across all grades was 87.5 (61.2) days. A total of three febrile neutropenia events were experienced among 3 patients (2%).

**Table 3:** Description of neutropenia events

Category, *n* (%)	P + AI (N = 85)	P + Ful (N = 54)	Overall (N = 139)
Number of events, number and (%) of patients with neutropenia onset by cycle			
Cycle 1	41, 30 (35%)	24, 22 (41%)	65, 52 (37%)
Cycle 2	15, 13 (15%)	14, 12 (22%)	29, 25 (18%)
Cycle 3	9, 8 (9%)	10, 8 (15%)	19, 16 (12%)
Cycle 4	10, 7 (8%)	4, 4 (7%)	14, 11 (8%)
Cycle 5	13, 7 (8%)	2, 2 (4%)	15, 9 (6%)
Cycle 6	1, 1 (1%)	2, 2 (4%)	3, 3 (2%)
Severity of neutropenia (number of events, number and (%) of patients)^a^			
Grade 1	27, 17 (20%)	16, 7 (13%)	43, 24 (17%)
Grade 2	34, 22 (26%)	21, 15 (28%)	55, 37 (27%)
Grade 3	29, 18 (21%)	21, 16 (30%)	50, 34 (24%)
Grade 4	3, 3 (4%)	0, 0 (0%)	3, 3 (2%)
Number of events, number and (%) of patients with dose adjustment or treatment interruption due to neutropenia	26, 18 (21%)	19, 14 (26%)	45, 32 (23%)
Dose adjustment	7, 6 (7%)	9, 9 (17%)	16, 15 (11%)
Treatment interruption	19, 13 (15%)	10, 9 (17%)	29, 22 (16%)

^a^As graded by investigators per CTCAE

### Daily pain

**Fig. 2:**
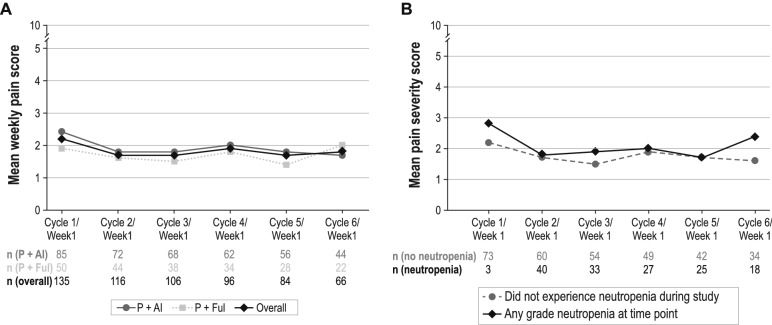
**a** Mean pain severity by cycle. **b** Mean Pain Severity by Cycle and Neutropenia Status *P* + *AI* palbociclib in combination with an aromatase inhibitor, *P* + *Ful* palbociclib in combination with fulvestrant

For patients overall, mean (SD) daily level of pain was 2.2 (2.31) on the NRS (0–10, with 10 being the worst possible pain), averaged across week 1 of cycle 1. At week 1 of cycle 6, mean (SD) pain was slightly lower at 1.8 (2.19). There were no substantial changes through week 1 of cycle 6 among the two palbociclib treatment groups (Fig. 2a). For patients experiencing neutropenia at the timepoint, mean (SD) pain ranged from 1.8 (2.2) to 2.8 (2.0) across the first week of each cycle and was slightly higher at cycle 3 and cycle 6 than among those not experiencing neutropenia at any time during the study (Fig. 2b).

### Daily fatigue

**Fig. 3:**
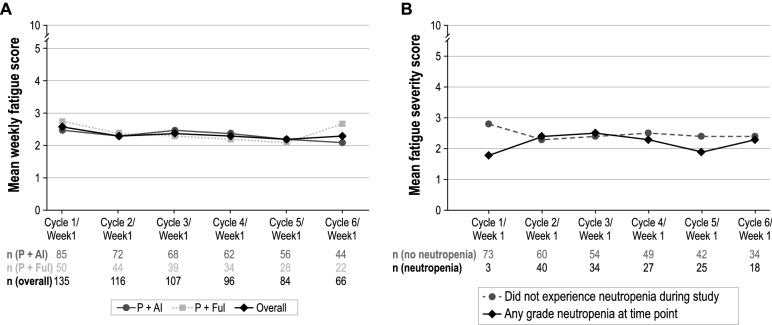
**a** Mean Fatigue Severity by Cycle. **b** Mean Fatigue Severity by Cycle and Neutropenia Status. *P* + *AI* = palbociclib in combination with an aromatase inhibitor; *P* + *Ful* palbociclib in combination with fulvestrant

Overall, mean (SD) daily level of fatigue was 2.7 (2.3) on the NRS (0–10, with 10 being the worst possible fatigue), averaged across week 1 of cycle 1. No substantial changes in fatigue NRS were observed through week 1 of cycle 6 overall or between the two palbociclib combination treatment groups (Fig. 3a). Despite slightly higher mean fatigue NRS scores among patients with no neutropenia events relative to those who experienced neutropenia at week 1 of cycle 1, no substantial differences between these groups were observed through week 1 of cycles 2–6 (Fig. 3b).

### Weekly mood

At the first week of cycle 1, a majority (90%) of participants reported their mood ranged from “Neutral” to “Most happy” (Figure S1). There were no substantial changes through week 1 of cycle 6 overall or among the two palbociclib treatment subgroups.

For patients without an indication of depression (i.e., CES-D-10 score < 10) at cycle 1, week 1, the mean percentage of nonmissing days where mood was ranked as “Most Happy,” “Happy,” or “Content” was 62% versus 24% in patients with depression. For patients without depression, the mean percentage of nonmissing days with a positive mood was stable across the first week of each cycle, while it ranged from 15 to 38% for patients with depression (Figure S2).

**Fig. 4:**
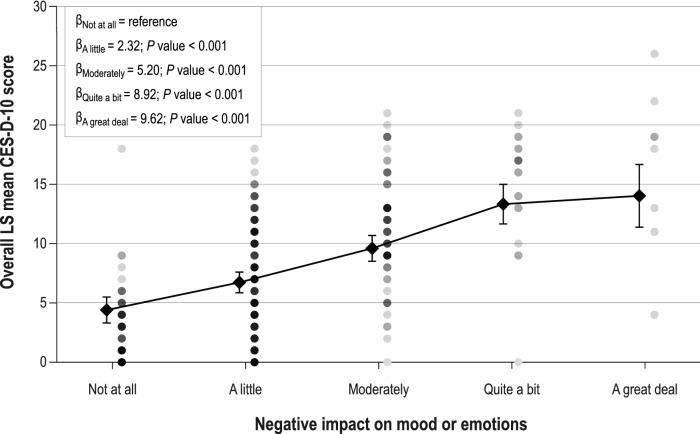
Relationship between weekly question of breast cancer’s negative impact on mood or Emotions and CES-D-10 Score CES-D-10 = 10-Item Center for Epidemiological Studies Depression Scale; LS = least squares. Circles represent actual values across cycles; diamonds represent LS mean estimates with confidence interval bars

The use of a mixed model for repeated measures, with fixed effects for negative impact on mood or emotions, suggests patients with mood or emotions not adversely impacted by breast cancer reported lower scores on the CES-D-10 (i.e., less indicative of depression). Specifically, the CES-D-10 least-squares mean (95% CI) for patients reporting “Not at all” was 4.41 (3.2–5.6), whereas the CES-D-10 least-squares mean for patients reporting “A great deal” of impact was 13.6 (11.0–16.3) (Fig. 4).

**Fig. 5:**
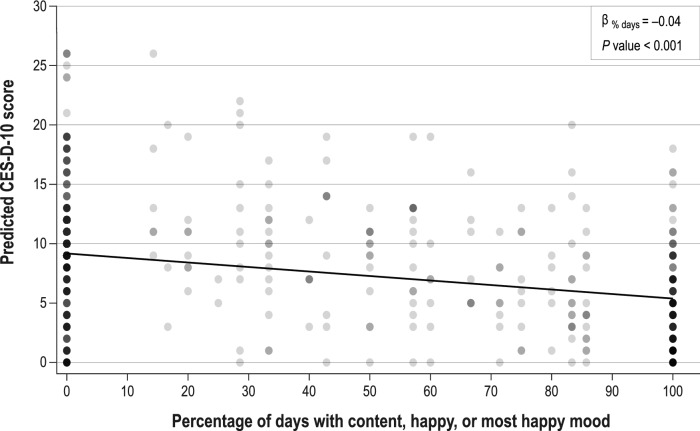
Relationship Between percentage of days in a week of content, happy, or most happy and CES-D-10 score CES-D-10 = 10-Item Center for Epidemiological Studies Depression Scale. Circles represent actual values across cycles

The use of a mixed model for repeated measures, with fixed effects for percentage of days with content, happy, or most happy mood, indicated that happy mood is associated with lower scores on the CES-D-10 (i.e., less indicative of depression): β = –0.04 (*P* < 0.001) (Fig. 5).

Descriptive analysis revealed no substantial difference in weekly mean percentage of nonmissing days for instances in which mood was ranked as either “Most happy,” “Happy,” or “Content” between patients who did or did not experience neutropenia.

### Interference from breast cancer or its treatment

Most patients across the first week of all cycles indicated aBC/mBC or its treatment interfered “Not at all” or “A little” with family life (Figure S3a) or social life (Figure S3b). Similarly, most patients indicated aBC/mBC or its treatment limited physical activity (Figure S4a), energy or stamina (Figure S4b), and productivity (Figure S4c) “Not at all” or “A little.” The percentage of patients who indicated aBC/mBC or its treatment limited these items “Quite a bit” or “A great deal” did not exceed 20% across the first week of all cycles and, in general, decreased from baseline. These findings were generally similar regardless of neutropenia status.

### SF-12

**Fig. 6:**
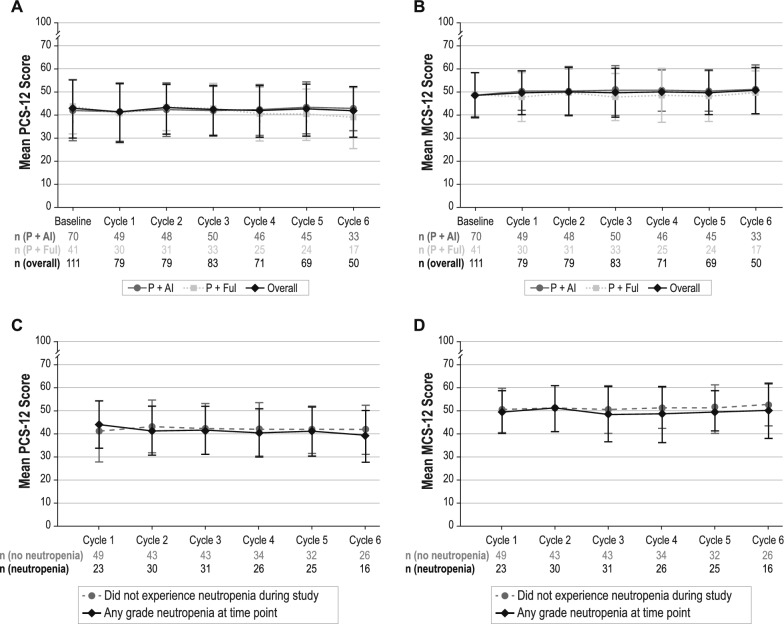
**a** Mean PCS-12 Score by Cycle. **b** Mean MCS-12 Score by Cycle. **c** Mean PCS-12 Score by Cycle and Neutropenia Status. **d** Mean MCS-12 Score by Cycle and Neutropenia Status. *MCS* Mental Component Summary, *P* + *AI* palbociclib in combination with an aromatase inhibitor, *P* + *Ful* palbociclib in combination with fulvestrant, *PCS* Physical Component Summary, *SF-12* 12-Item Short Form Health Survey, Bars represent standard deviation

Descriptive examination of the data from baseline through cycle 6 indicated that MCS-12 and PCS-12 scores were generally stable and indicated good physical and mental functioning among patients. At baseline, PCS-12 mean (SD) scores were 42.5 (12.5) overall, 42.0 (13.0) for the P + AI subgroup, and 41.3 (10.9) for the P + Ful subgroup (Fig. 6a); mean (SD) MCS-12 scores were 48.8 (9.7), 48.6 (9.9), and 49.9 (9.6), respectively (Fig. 6b). No substantial changes from baseline were observed in mean SF-12 scores overall or among the two different palbociclib combination therapies.

Based on the mixed models for repeated measures, the least-squares mean (95% CI) changes from baseline across all cycles were −0.5 (− 1.8 to 0.9) and 0.7 (− 0.6 to 1.9) for the PCS-12 and MCS-12, respectively. Least-squares mean changes from baseline PCS-12 and MCS-12 scores across all cycles were 0.2 (− 1.5 to 2.0) and 1.4 (− 0.1 to 3.0) for P + AI patients versus − 1.0 (− 3.2 to 1.2) and − 0.5 (− 2.5 to 1.5) for P + Ful patients.

Descriptive examination of the mean (SD) PCS-12 scores by neutropenia status indicated patients with neutropenia at cycle 6 had a score of 39.0 (11.3) compared with 41.8 (10.63) for those who did not experience neutropenia during the study (Fig. 6c). For the MCS-12, the mean (SD) scores at cycle 6 were 50.0 (12.1) and 52.5 (9.1), respectively, for patients with neutropenia at cycle 6 and those without a neutropenia event during the study (Fig. 6d). Based on the mixed models for repeated measures, the least-squares mean (95% CI) change from baseline across all cycles for patients with and without neutropenia was 0.3 (− 2.1 to 1.6) and -0.6 (− 2.1 to 1.0) for the PCS-12 and − 0.3 (− 2.1 to 1.6) and 0.9 (− 0.6 to 2.3) for the MCS-12.

### CES-D-10

**Fig. 7:**
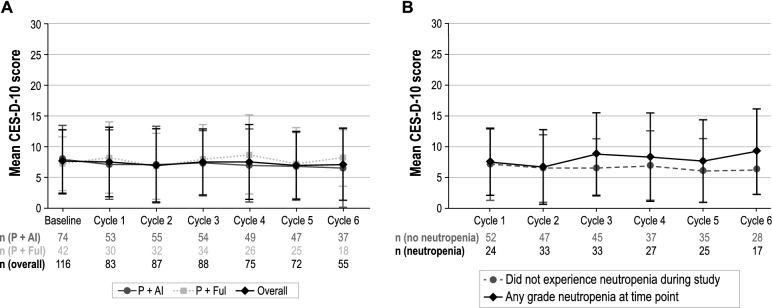
**a** Mean CES-D-10 Score by Cycle. **b** Mean CES-D-10 by Cycle and Neutropenia Status *CES-D-10* 10-Item Center for Epidemiological Studies Depression Scale, *MCS* = Mental Component Summary, *P* + *AI* palbociclib in combination with an aromatase inhibitor, *P* + *Ful* palbociclib in combination with fulvestrant, Bars represent standard deviation

Descriptive examination of CES-D-10 scores from baseline through cycle 6 indicated mean scores overall and in both palbociclib combination subgroups were < 10, the score threshold considered to indicate the presence of depression (Fig. 7a). At baseline, mean (SD) CES-D-10 scores were 7.6 (5.2) overall, 7.9 (5.6) for P + AI, and 7.2 (4.4) for P + Ful. At cycle 6, mean (SD) CES-D-10 scores were 6.5 (6.4) and 8.2 (4.6) for P + AI and P + Ful, respectively. Overall, mean (SD) change from baseline in CES-D-10 score was 0.5 (4.9) at cycle 6.

Based on the mixed models for repeated measures, the least-squares mean (95% CI) change from baseline across all cycles was 0.0 (− 0.7 to 0.6). Least-squares mean changes from baseline were consistently lower in P + AI patients than in P + Ful patients. At cycle 6, least-squares mean changes from baseline were − 0.4 (− 1.2 to 0.5) and 0.5 (− 0.6 to 1.6), respectively, for P + AI and P + Ful.

Descriptive examination of the mean (SD) CES-D-10 scores by neutropenia status indicated patients with neutropenia at cycle 6 had a score of 9.2 (6.9) compared with 6.2 (5.6) for those who did not experience neutropenia during the study (Fig. 7b). Based on the mixed models for repeated measures, the least-squares mean (95% CI) change from baseline across all cycles for patients with neutropenia and those without neutropenia was 0.8 (− 0.1 to 1.8) and − 0.3 (− 1.1 to 0.4), respectively.

### Overall weekly health rating and quality of life

Most patients across the first week of all cycles indicated their overall health rating and QOL was “Good,” “Very good,” or “Excellent” (Figure S5a and S5b). In the first week across all cycles, weekly overall health and QOL was ranked as “Poor” by less than 8% and 4% of patients, respectively. These findings were generally similar regardless of neutropenia status, where ratings remained stable across cycles.

## Discussion

Treatment for HR + /HER2 − aBC/mBC has evolved with the approval of the CDK4/6 inhibitor class combined with endocrine agents, beginning with palbociclib in February 2015 [20]. Real-world data regarding patient QOL and experiences while receiving palbociclib, however, are limited.

To our knowledge, our study is one of the first to evaluate the day-to-day impacts of aBC/mBC and its treatment on patients and the effects of treatment-induced neutropenia on patients’ daily functioning outside the context of clinical trials. We found that, on the whole, patients treated with palbociclib combination therapy experienced low levels of baseline pain and fatigue, which remained stable across cycles; general health status as measured by the SF-12 remained consistent throughout treatment and was generally consistent with published norms for individuals diagnosed with cancer (excluding skin cancer), and the presence of depression, as indicated by the patient-reported CES-D-10, was low and did not change substantially over time [18]. Patients, on average, reported a neutral or positive mood. Patient-reported QOL and overall health was primarily “Good,” “Very good,” or “Excellent.” Patients with neutropenia, on the whole, did not show decreases in QOL during the study, and findings were consistent with those who did not experience neutropenia.

With limited real-world information available regarding patient experiences with aBC/mBC and treatment-induced neutropenia, this study provides valuable data to inform patient and physician treatment discussions and health care decision-making.

## Limitations

The sample of participating sites was a convenience sample and may not be representative of all US centers treating patients with aBC/mBC. Only 139 evaluable patients of a targeted 300 were enrolled. The patient selection and monitoring procedures are those applied per the treating physician’s usual treatment paradigm and not dictated by the protocol. Heterogeneous patient populations could make the interpretation of the outcomes difficult.

As with all studies requiring patients to self-report outcomes and behavior, completeness and accuracy of reporting can be a concern. AE collection was limited to what was spontaneously disclosed during standard of care visits and retrospective medical record review. Patients without a smartphone were not eligible for participation and may have different outcomes. Some patients may have discontinued the study early because of progression or declining health, which is associated with QOL. Early withdrawals decreased sample sizes at later cycles but examination of PRO assessments by completion status found no substantial differences. Correlation of disease burden with severity of pain was not examined in this study. Results of this study are subject to potential selection bias and responder bias, and whether individuals who elected not to participate would have reported different outcomes is unknown.

## Conclusion

This study demonstrated that palbociclib-treated patients, on average, reported consistently low levels of pain and fatigue as well as good QOL, and overall health remained stable during 6 months of treatment. Episodes of neutropenia did not impact QOL.

## Electronic supplementary material

Below is the link to the electronic supplementary material.

Electronic supplementary material 1 (DOC 738 kb)

## Data Availability

The datasets generated during and/or analyzed during the current study are available from the corresponding author on reasonable request.

## Footnotes

1 Initially, palbociclib with letrozole until the indication for palbociclib was broadened in March 2017.

## References

[1] Surveillance, Epidemiology, and End Results Program. SEER cancer stat facts: female breast cancer. National Cancer Institute. Bethesda, MD. https://seer.cancer.gov/statfacts/html/breast.html. Accessed 1 June 2020.

[2] PDQ Adult Treatment Editorial Board. PDQ breast cancer treatment (adult). Bethesda, MD: National Cancer Institute. https://www.cancer.gov/types/breast/hp/breast-treatment-pdq. Accessed 1 June 2020.

[3] Ibrance (palbociclib) FDA prescribing information (Sept 2019). https://www.accessdata.fda.gov/drugsatfda_docs/label/2019/207103Orig1s012lbl.pdf. Accessed 1 June 2020.

[4] Finn RS, Crown JP, Lang I, et al (2015) The cyclin-dependent kinase 4/6 inhibitor palbociclib in combination with letrozole versus letrozole alone as first-line treatment of oestrogen receptor-positive, HER2-negative, advanced breast cancer (PALOMA-1/TRIO-18): a randomized phase 2 study. Lancet 16(1):25–35. doi: 10.1016/S1470-2045(14)71159-3. http://www.thelancet.com/pdfs/journals/lanonc/PIIS1470-2045(14)71159-3.pdf. 10.1016/S1470-2045(14)71159-3 25524798

[5] Finn RS, Martin M, Rugo HS, et al (2016) PALOMA-2: primary results from a phase III trial of palbociclib (P) with letrozole (L) compared with letrozole alone in postmenopausal women with ER+/HER2− advanced breast cancer (aBC). J Clin Oncol 34(suppl; abstract 507).

[6] Rugo HS, Finn RS, Diéras V (2019). Palbociclib plus letrozole as first-line therapy in estrogen receptor-positive/human epidermal growth factor receptor 2-negative advanced breast cancer with extended follow-up. Breast Cancer Res Treat.

[7] DeMichele A, Cristofanilli M, Brufsky A, et al (2019) Overall survival for first-line palbociclib plus letrozole vs letrozole alone for HR+/HER2− metastatic breast cancer patients in US real-world clinical practice. Presented at the 2019 San Antonio Breast Cancer Symposium; December 10–14; San Antonio, Texas. Abstract P1–19–02.

[8] Cristofanilli M, Turner NC, Bondarenko I (2016). Fulvestrant plus palbociclib versus fulvestrant plus placebo for treatment of hormone-receptor-positive, HER2−negative metastatic breast cancer that progressed on previous endocrine therapy (PALOMA-3): final analysis of the multicentre, double-blind, phase 3 randomised controlled trial. Lancet Oncol.

[9] Kisqali (ribociclib) FDA prescribing information (July 2020) https://www.accessdata.fda.gov/drugsatfda_docs/label/2020/209092s005lbl.pdf. Accessed 15 July 2020.

[10] Verzenio (abemaciclib) FDA prescribing information (March 2020) https://www.accessdata.fda.gov/drugsatfda_docs/label/2020/208716s004lbl.pdf. Accessed 15 July 2020.

[11] Cardoso F, Senkus E, Costa A (2018). 4th ESO-ESMO International Consensus Guidelines for Advanced Breast Cancer (ABC 4). Ann Oncol.

[12] Rugo HS, Rumble RB, Macrae E (2016). Endocrine therapy for hormone receptor-positive metastatic breast cancer: American Society of Clinical Oncology Guideline. J Clin Oncol.

[13] Ibrance (palbociclib) FDA prescribing information (March 2017) https://www.accessdata.fda.gov/drugsatfda_docs/label/2017/207103s004lbl.pdf. Accessed 1 June 2020.

[14] Björgvinsson T, Kertz SJ, Bigda-Peyton JS (2013). Psychometric properties of the CES-D-10 in a psychiatric sample. Assessment.

[15] Ware JE, Kosinski M, Keller SD (1996). A 12-Item Short-Form Health Survey: construction of scales and preliminary tests of reliability and validity. Med Care.

[16] Jenkinson C, Layte R, Jenkinson D (1997). A shorter form health survey: can the SF-12 replicate results from the SF-36 in longitudinal studies?. J Public Health.

[17] Irwin M, Artin K, Oxman MN (1999). Screening for depression in the older adult: criterion validity of the 10-Item Center for Epidemiological Studies Depression Scale (CES-D). Arch Intern Med.

[18] Pew Research Center (2017) Questionnaire design. http://www.pewresearch.org/methodology/u-s-survey-research/questionnaire-design/. Accessed 1 June 2020.

[19] Ware JE, Kosinski M, Turner-Bowker DM (2002). How to score Version 2 of the SF-12^®^ Health Survey (With a Supplement Documenting Version 1).

[20] FDA (2015) Accelerated approval of new drug application for Ibrance (palbociclib). https://www.accessdata.fda.gov/drugsatfda_docs/appletter/2015/207103orig1s000ltr.pdf. Accessed 1 June 2020.

