# Supplementary material for: A prospective observational study of patient-reported functioning and quality of life in advanced and metastatic breast cancer utilizing a novel mobile application
Source: Breast Cancer Res Treat. 2021 Jan 11;187(1):113–24. doi: 10.1007/s10549-020-06082-7 (PMC8062359; doi:10.1007/s10549-020-06082-7)

# Online Resources

A prospective observational study of patient-reported functioning and quality of life in advanced and metastatic breast cancer utilizing a novel mobile application

Breast Cancer Research and Treatment

David Richardson^1^, Lin Zhan^2^, Reshma Mahtani^3^, Lynn McRoy^2^, Debanjali Mitra^2^, Maria Reynolds^1^, Dawn Odom^1^, Kelly Hollis^1^, James A. Kaye^1^, Cheryl Jones^4^, Jeffrey Hargis^5^

^1^RTI Health Solutions, Research Triangle Park, NC

^2^Pfizer, Inc., New York, NY

^3^University of Miami, Deerfield Beach, FL

^4^Northside Hospital Inc., Atlanta, GA,

^5^Norton Cancer Institute, Louisville, KY

Corresponding Author:

David Richardson (ORCID# 0000-0002-6481-8851); E-mail: drichardson@rti.org

Online Resource 1, Figure S1. Mean Percentage of Days Reported for Each Mood Category


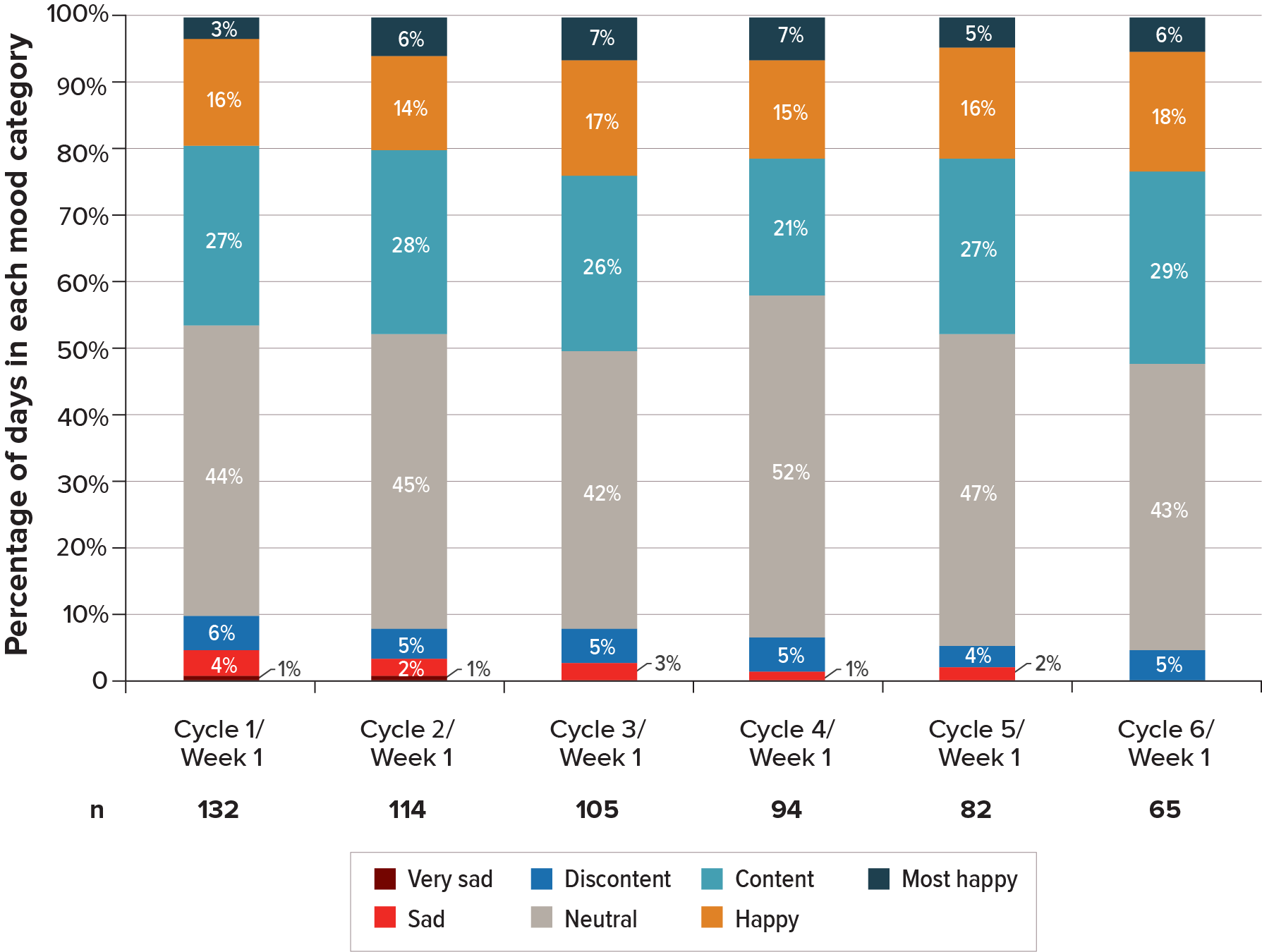


Online Resource 2, Figure S2. Relationship Between Mean Percentage of Days Reported for Each Mood Category and CES-D-10 Score


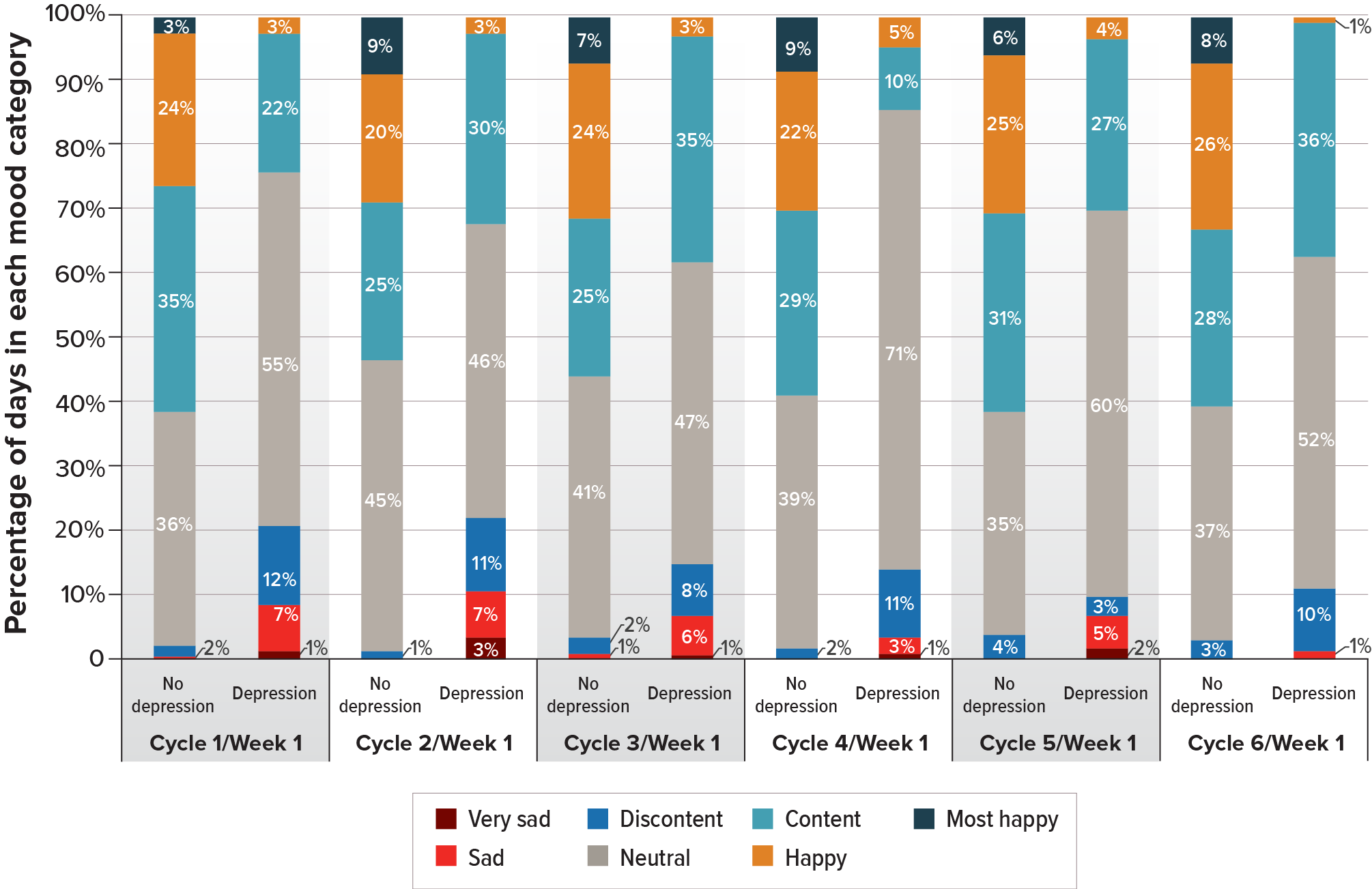


Online Resource 3, Figure S3a. Weekly Interference With Family Life Caused by aBC/mBC or Its Treatment


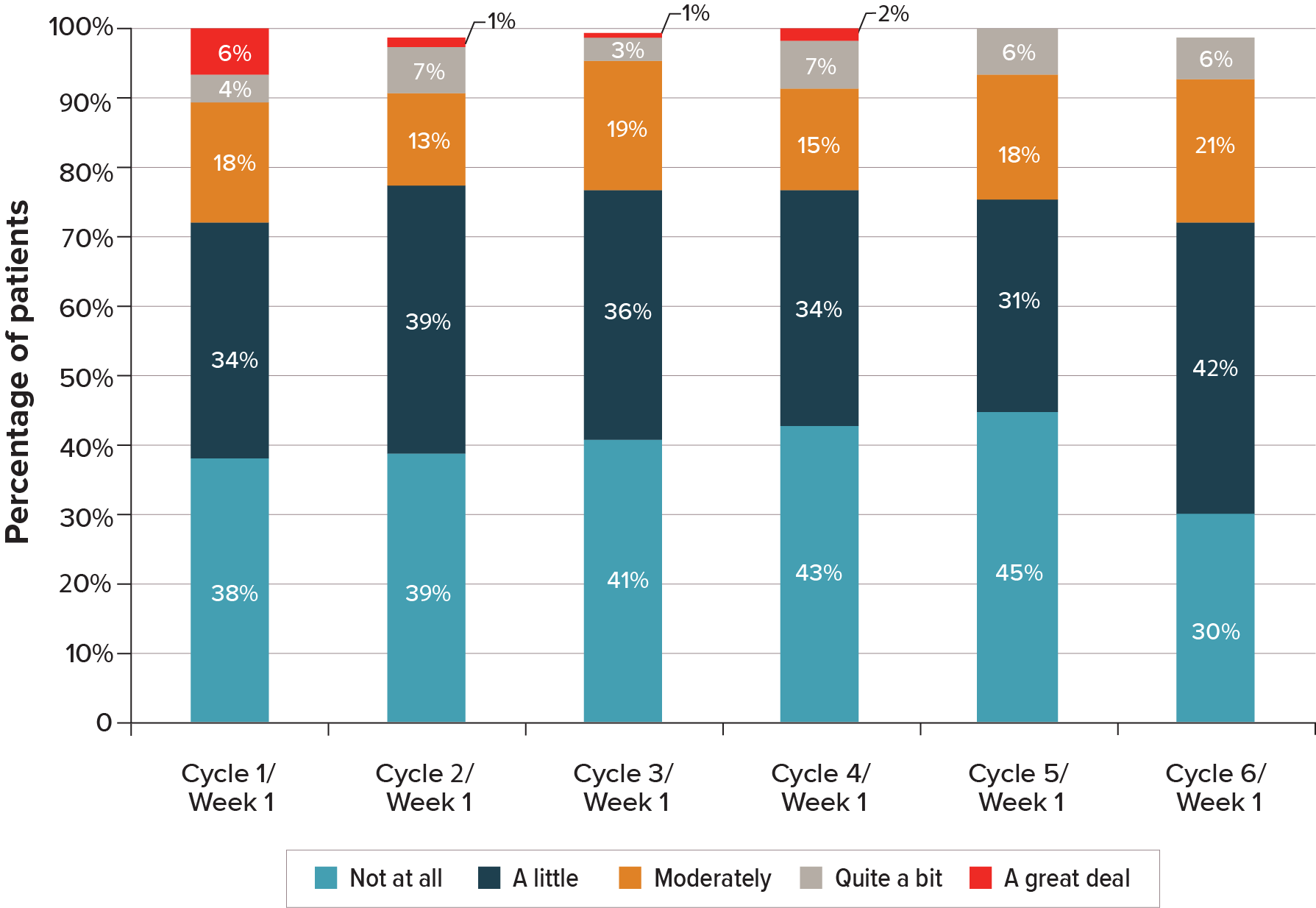


Online Resource 3, Figure S3b. Weekly Interference With Social Life Caused by aBC/mBC or Its Treatment


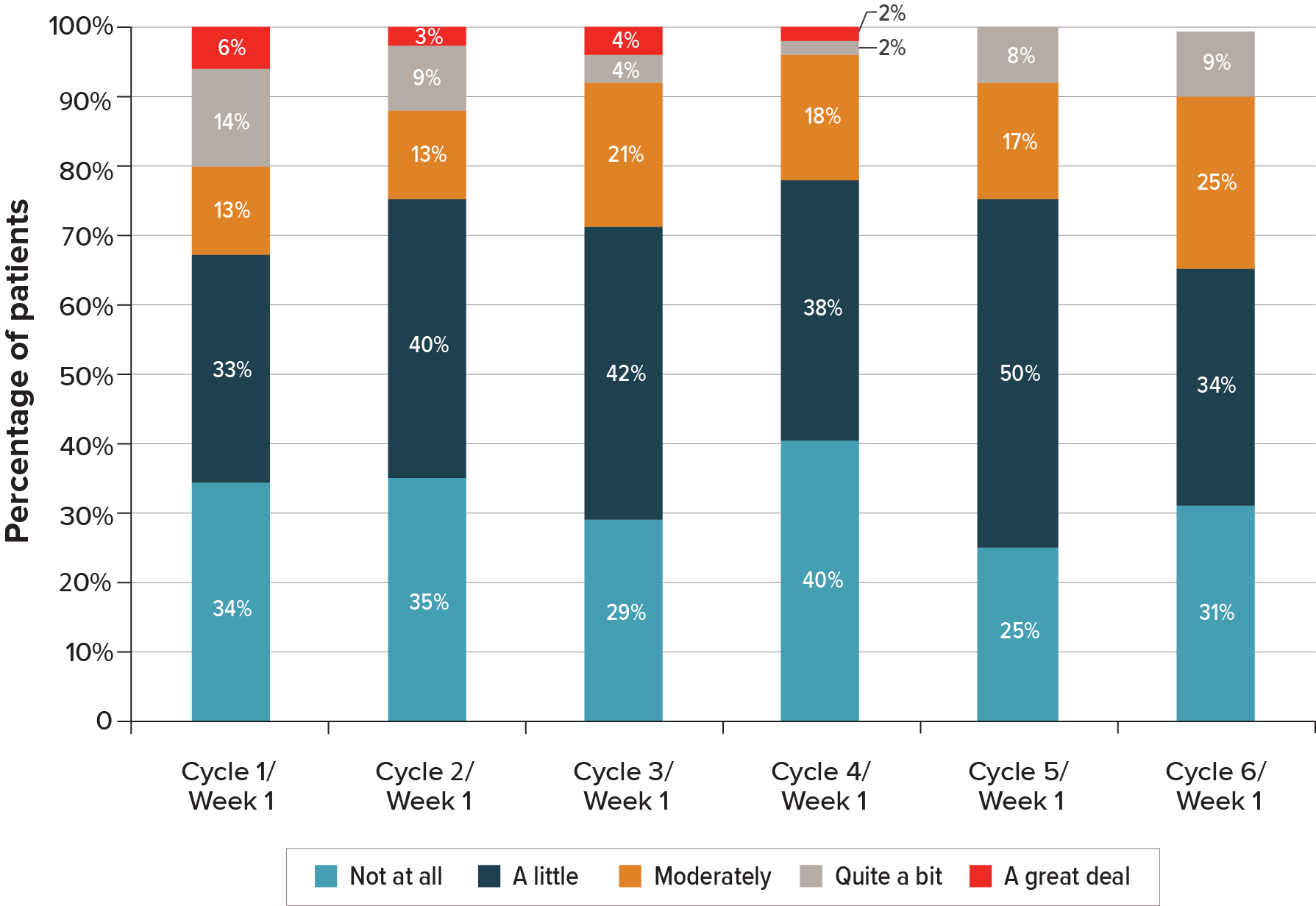


aBC = advanced breast cancer; mBC = metastatic breast cancer.

Online Resource 4, Figure S4a. Weekly Limitation on Physical Activity Caused by aBC/mBC or Its Treatment


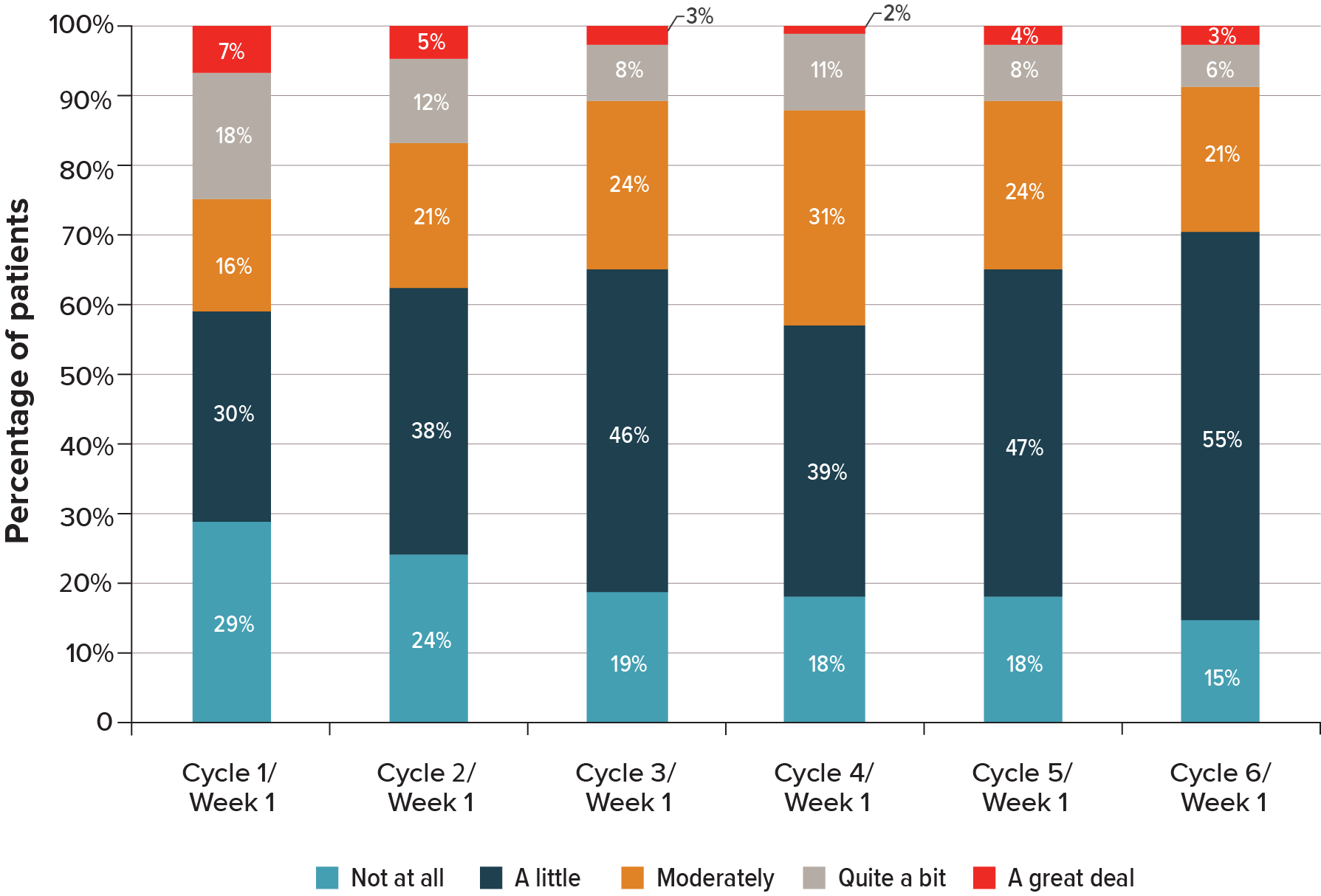


Online Resource 4, Figure S4b. Weekly Limitation on Energy or Stamina Caused by aBC/mBC or Its Treatment


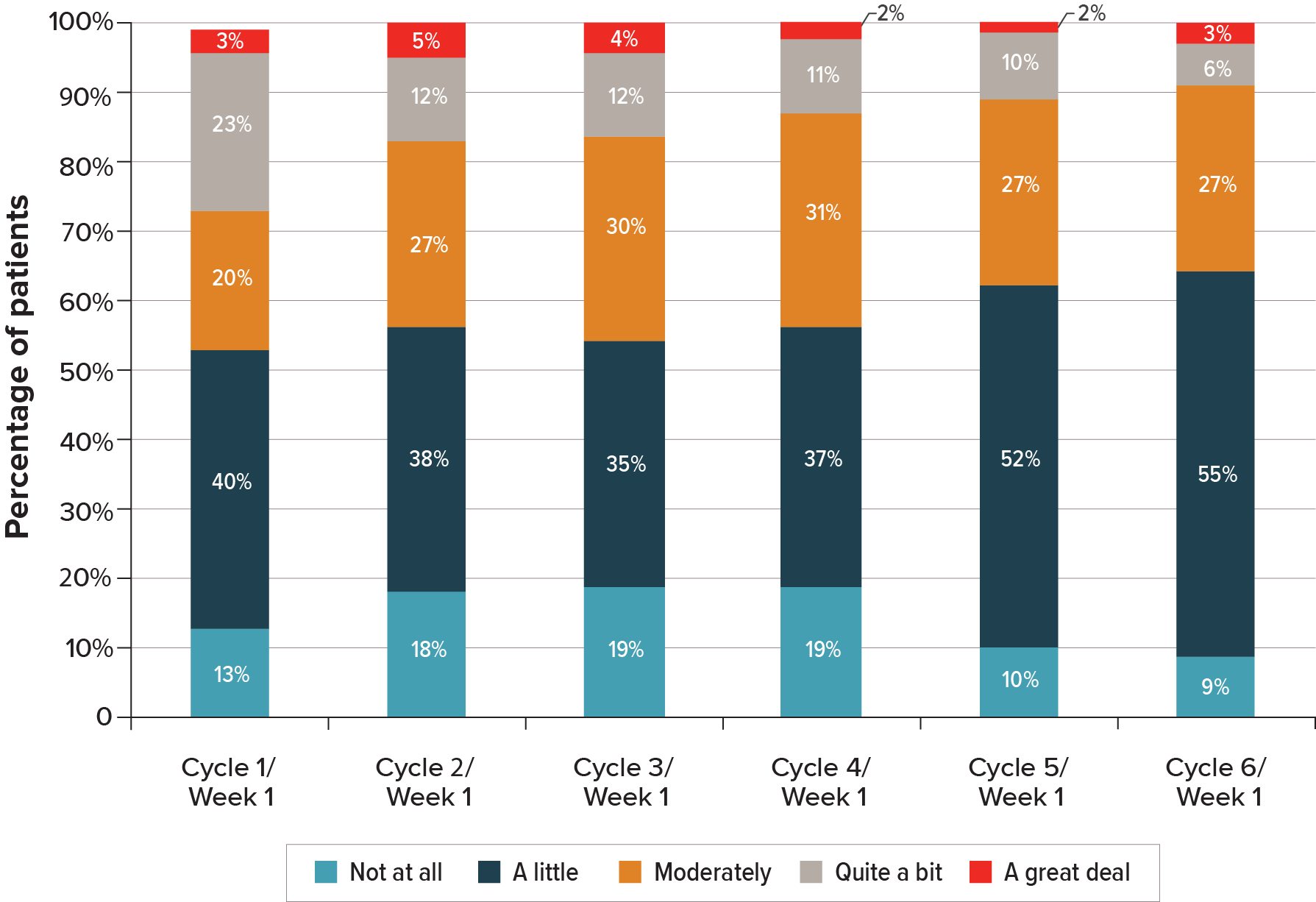


Online Resource 4, Figure S4c. Weekly Limitation on Productivity Caused by aBC/mBC or Its Treatment


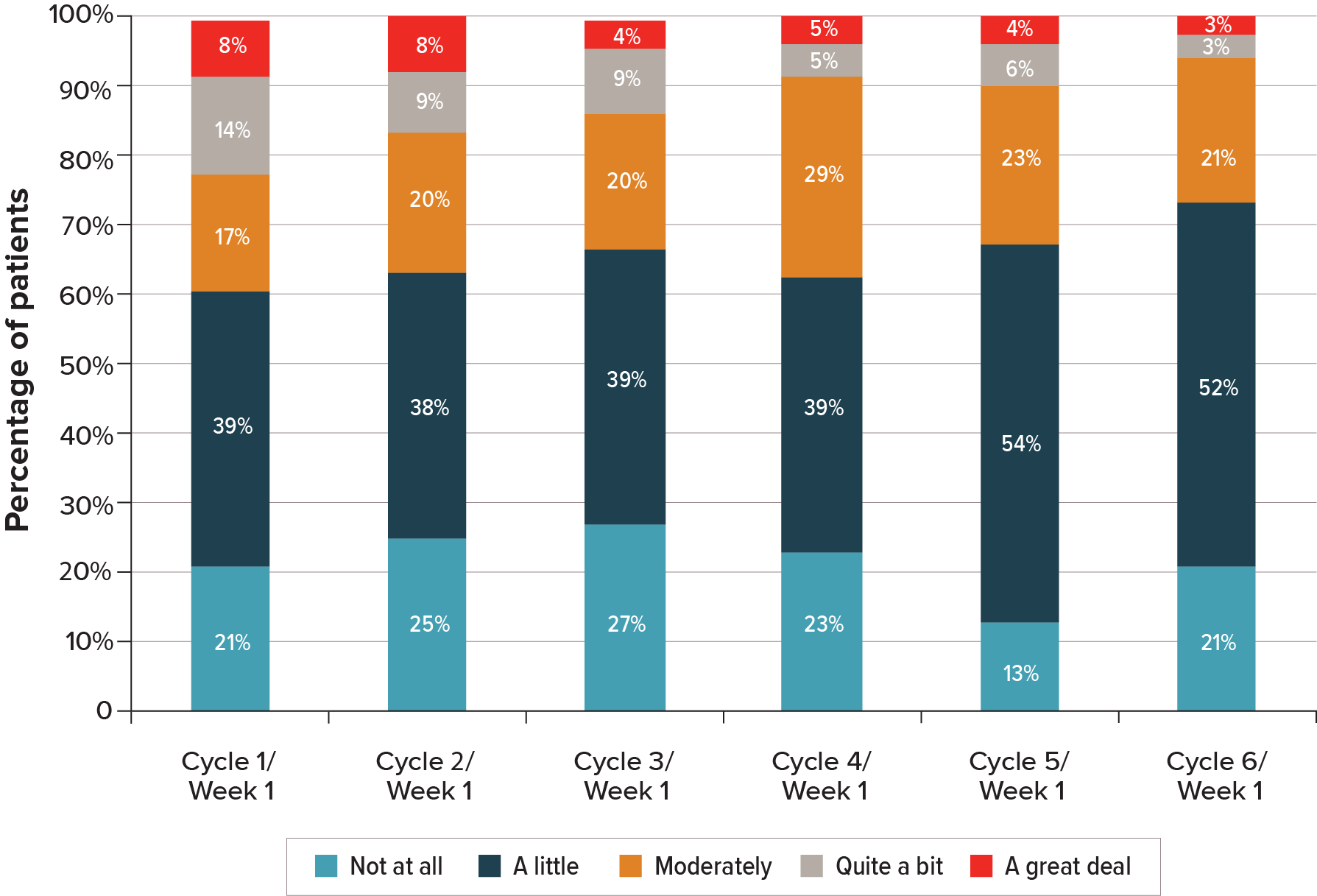


aBC = advanced breast cancer; mBC = metastatic breast cancer

Online Resource 5, Figure S5a. Overall Weekly Health Rating


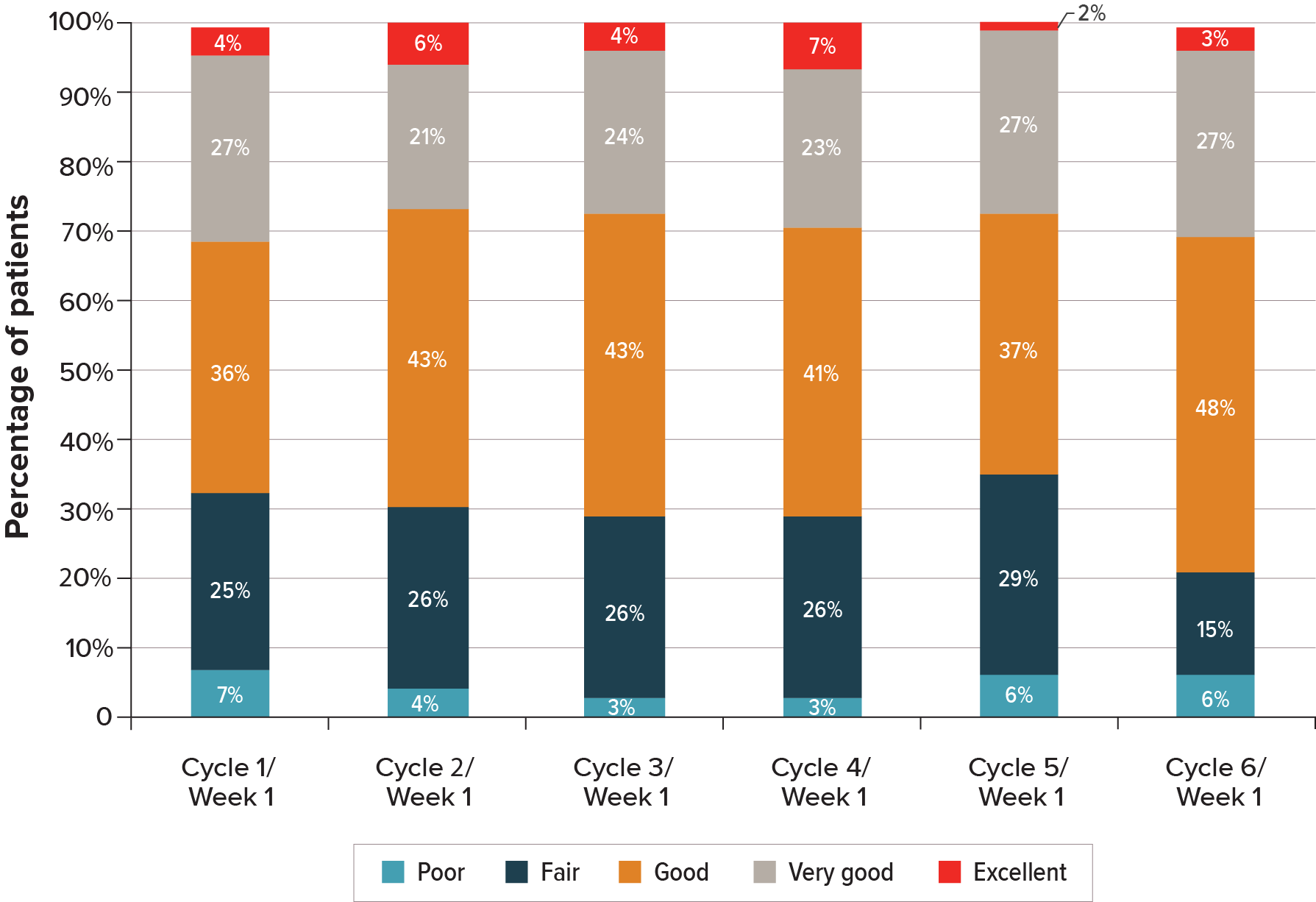


Online Resource 5, Figure S5b. Overall Weekly Quality of Life


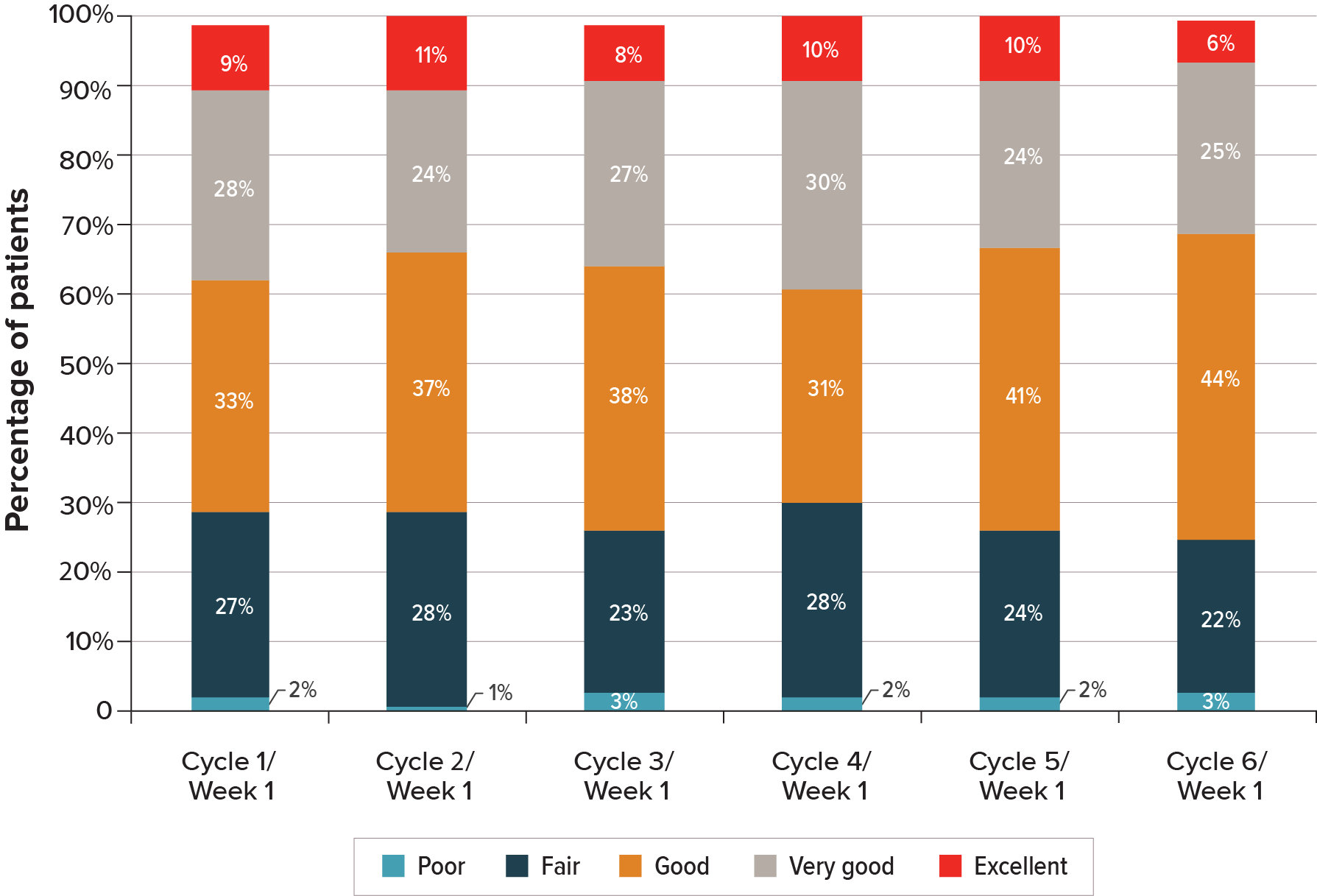

Supplement: Supplementary file 1 — Electronic supplementary material 1 (DOC 738 kb) [file 10549_2020_6082_MOESM1_ESM.doc]
